# Prognostic Significance of Serial Ultrasonography in Placenta Accreta Spectrum and Its Impact on Perinatal Outcomes

**DOI:** 10.3390/medicina61091612

**Published:** 2025-09-05

**Authors:** Antonia Varthaliti, Alexandros Psarris, Pelopidas Koutroumanis, Giwrgos Gkiaourakis, Maria Anastasia Daskalaki, Panos Antsaklis, George Daskalakis, Marianna Theodora

**Affiliations:** 1st Department of Obstetrics and Gynecology, “Alexandra” General Hospital, National and Kapodistrian University of Athens, 80 Vasilissis Sofias Avenue, 11528 Athens, Greece; psarris.alexandros@gmail.com (A.P.); pelejk@hotmail.com (P.K.); gkiaourakisgeo@gmail.com (G.G.); anastasia.daskalaki00@gmail.com (M.A.D.); panosant@gmail.com (P.A.); gdaskalakis@yahoo.com (G.D.)

**Keywords:** placenta accreta spectrum, ultrasound, perinatal, outcomes, obstetrics

## Abstract

Placenta accreta spectrum (PAS) disorders remain a major cause of maternal morbidity and adverse perinatal outcomes due to abnormal placental adherence and invasion. Early and accurate prenatal diagnosis is essential to optimize surgical planning and reduce complications. Although ultrasound is well established as the cornerstone for PAS detection, the potential role of serial ultrasonography in refining risk assessment and predicting outcomes is increasingly being explored. Monitoring with serial ultrasonographic imaging may offer valuable insights into the progression of sonographic features, such as placental lacunae, myometrial thinning, placental bulge, and bladder wall disruption, which can predict surgical complexity and perinatal risk and influence decision-making and management. However, there is still limited evidence about the prognostic value of serial scans, and the variability in interpreting ultrasound markers continues, presenting challenges. While scoring systems incorporating ultrasound features show promise for risk stratification, further validation in larger studies is needed. Future research should focus on standardizing ultrasound protocols, validating predictive models, and exploring technological innovations, including artificial intelligence, to enhance diagnostic precision. Incorporating serial ultrasound assessments thoughtfully into clinical practice may improve individualized care and outcomes for women affected by PAS, but more studies are required.

## 1. Introduction

Placenta accreta spectrum (PAS) is a significant obstetric complication characterized by abnormal placental adherence or invasion into the uterine wall, which may lead to obstetric hemorrhage, shock, and severe maternal morbidity and mortality [1]. The incidence of PAS has risen dramatically in parallel with global cesarean delivery rates, now occurring in approximately 1 in 533 deliveries. Prior cesarean delivery, particularly in the setting of placenta previa, is the strongest risk factor, with advanced maternal age, multiparity, and prior uterine surgery contributing additional risk.

The terminology and classification of PAS have evolved considerably over time. The entity was first described by Irving and Hertig in 1937 as abnormal placental attachment directly to the myometrium without intervening decidua [2]. Later refinements introduced distinctions based on the depth of placental invasion: placenta accreta (superficial adherence to myometrium), increta (invasion into myometrium), and percreta (penetration through the uterine serosa, potentially invading adjacent organs). Collectively, these conditions are now unified under the term placenta accreta spectrum, as endorsed by international guidelines such as those from FIGO, which also provided a detailed classification integrating clinical and histopathological criteria [3,4].

Accurate antenatal diagnosis of PAS is critical to improving maternal and neonatal outcomes. Ultrasonography has become the primary imaging modality due to its accessibility, safety, and cost-effectiveness [5]. Characteristic ultrasound findings include multiple placental lacunae, loss of the retroplacental clear zone, myometrial thinning, bladder wall interruption, and abnormal uterovesical vascularity. Recent studies show that ultrasound achieves high diagnostic accuracy in PAS, with pooled sensitivity and specificity of approximately 87% and 86%, respectively [6]. However, diagnostic performance varies depending on placental location, sonographic criteria used, and operator experience.

Despite these advances, current research predominantly focuses on single time-point ultrasound assessments, typically in the second or third trimester [7]. Given that PAS is a dynamic process, evolving as pregnancy progresses, there is increasing interest in whether serial ultrasonographic evaluations may yield superior prognostic information. Emerging evidence suggests that progressive changes in ultrasound markers, such as increasing vascular lacunae, deepening myometrial thinning, or expanding hypervascularity, may correlate with the severity of invasion and subsequent surgical complexity, hemorrhage risk, and neonatal outcomes.

The American College of Obstetricians and Gynecologists, the Society for Maternal-Fetal Medicine, and the Society of Gynecologic Oncology recommend that ultrasound should be performed in women with risk factors for PAS (such as placenta previa and prior cesarean delivery), with most guidelines favoring second-trimester screening and some suggesting initiation in the first trimester [8,9]. However, the optimal timing and frequency of serial ultrasound examinations remain unclear. Many physicians perform monthly ultrasounds, but this approach has not been proven to improve maternal or neonatal outcomes. A reasonable protocol, as described in the literature, is to perform ultrasound examinations at approximately 18–20, 28–30, and 32–34 weeks of gestation in asymptomatic patients [8]. This schedule allows for assessment of previa resolution, placental location, and possible bladder invasion, which are critical for planning delivery and surgical management.

Nevertheless, the prognostic significance of serial ultrasound assessments in PAS remains incompletely understood. Evidence is limited and heterogeneous regarding how temporal changes in imaging features relate to clinical decision-making and perinatal outcomes. Understanding these relationships is essential for refining risk stratification, guiding multidisciplinary management, and potentially reducing maternal and neonatal morbidity associated with this complex disorder.

This review aims to synthesize current evidence regarding the prognostic significance of serial ultrasonography in pregnancies complicated by PAS and its impact on perinatal outcomes and to provide evidence-based care recommendations for one of the most difficult problems in obstetrics by clarifying the correlation between evolving sonographic characteristics and clinical outcomes and disease severity.

## 2. Evaluation of PAS

### 2.1. Diagnostic Features

The primary imaging tool for antenatal assessment of placenta accreta spectrum, remains ultrasonography, which provides important information about the diagnosis and thus, risk assessment and surgery planning. PAS encompasses a range of abnormal placental adherence and invasion, from superficial attachment of the placenta to deep infiltration into or through the uterine wall and, in severe cases, invasion of surrounding pelvic organs [10]. The rising prevalence of PAS parallels the global increase in cesarean deliveries, making timely and accurate diagnosis essential to improving maternal and neonatal outcomes [4,8,11,12,13].

Classic sonographic signs of PAS have been well established over the last decades. One of the earliest and most frequently described features is the loss or irregularity of the hypoechoic retroplacental clear zone, which normally delineates the interface between the placenta and the myometrium. Its absence suggests abnormal placental implantation and invasion [12,14,15]. Another diagnostic sign is the presence of numerous, often irregular placental lacunae, that are vascular spaces within the placenta that have turbulent flow with color Doppler [16]. The number and size of these lacunae is essential, as extensive lacunae have been associated with deeper invasion and greater surgical risk [8,15,17].

Additional findings include focal interruptions or thinning of the myometrium beneath the placental bed, distortion or bulging of the uterine contour, and irregularities in the uterine serosa–bladder interface [8,13,14,18]. Color Doppler enhances the diagnostic accuracy because it reveals increased subplacental vascularity, bridging vessels traversing the uterine wall, and chaotic blood flow patterns within the placenta [8,14,19]. Three-dimensional power Doppler imaging may provide further detail, especially in suspected cases of placenta percreta, where hypervascular networks extend beyond the uterine serosa [14,15]. The diagnostic features are summarized in Table 1.

Despite advances in imaging, diagnostic accuracy varies depending on placental location, operator expertise, and the presence of previous uterine surgery. Recent studies report high pooled sensitivities and specificities for ultrasound in PAS, often exceeding 85–90%, but rates may be lower for posterior placentas or in low-resource settings [8,12,13,14,19]. Furthermore, the absence of ultrasonographic findings does not entirely exclude PAS, emphasizing the need to integrate clinical risk factors such as prior cesarean sections, placenta previa, and other uterine procedures [8,11,13].

It is essential to establish consistency in diagnosis, which has led to the development of standardized reporting protocols. Efforts are made by the expert groups, including the FIGO Placenta Accreta Spectrum Consensus Panel and the European Working Group on Abnormally Invasive Placenta, who have published consensus statements defining ultrasound markers and advocating for uniform terminology that all clinicians dealing with PAS should follow. Such standardization aims to reduce interobserver variability and improve diagnostic accuracy, particularly in centers with less experience in managing PAS [14,20,21].

### 2.2. Serial Ultrasonography

Most studies have focused on single-point ultrasound assessments; however, it is widely known that PAS is a dynamic condition with evolving features throughout gestation. Serial ultrasonography has emerged as a promising method for monitoring the progression of the disease, refining diagnostic certainty, and assessing the prognosis [12,13,14,17].

Serial ultrasounds are essential for tracking changes in important features, such as the progression of placental lacunae, the increasing myometrial thinning, and the expanding areas of abnormal vascularity. For example, studies have documented how minor abnormalities detected in mid-pregnancy can evolve into more definitive signs of PAS in the third trimester, highlighting the potential value of longitudinal assessments [15]. Chong et al. [17] have proposed an ultrasonic scoring system that incorporates parameters like placental position, thickness, clear zone integrity, bladder interface, and vascular characteristics. Higher cumulative scores in serial assessments were significantly correlated with increased intraoperative blood loss and higher rates of hysterectomy, emphasizing the prognostic potential of repeated evaluations [17,20,22].

However, there is still no standard protocol regarding the optimal timing, frequency, and interpretation of serial ultrasound scans in PAS. Current guidelines suggest serial assessments at specific gestational age, typically around 18–20, 28–30, and 32–34 weeks in high-risk pregnancies [8,12]. Additionally, there is a need of a consensus in order to evaluate the interpretation of subtle sonographic changes, particularly in distinguishing between adherent and invasive PAS forms, because there is substantial interobserver variability [8,13,14].

Clinical guidelines agree that integrating serial ultrasonography into PAS management may enhance patient counseling, multidisciplinary planning, and surgical readiness. Nevertheless, no imaging modality, including serial ultrasound, can definitively distinguish the depth of invasion or predict surgical complexity in all cases [11,21]. Prospective multicenter studies are needed in order to establish validated scoring systems and clear protocols for incorporating serial ultrasonography into the daily clinical practice and decision-making [13,14,17].

Despite current limitations, the evolving sonographic findings identified through serial imaging, especially progressive lacunae formation, worsening myometrial thinning, and increasing hypervascularity, are valuable indicators of the severity of the disease. These insights may allow earlier intervention and more precise surgical planning, ultimately contributing to improved maternal and neonatal outcomes in pregnancies complicated by PAS [8,12,13,14,17].

### 2.3. Role of Magnetic Resonance Imaging in PAS Diagnosis

While ultrasonography remains the basic imaging technique for the prenatal assessment of PAS, magnetic resonance imaging (MRI) has been used as an adjunct in clinical conditions where ultrasound findings are ambiguous. MRI offers excellent soft tissue contrast and can provide clearer delineation of the placenta’s relationship to surrounding pelvic structures, including the bladder and parametrial tissues [19,23,24,25]. It is found that MRI has comparable sensitivity and specificity to ultrasound for detecting PAS, but may offer advantages in posterior placentas where visualization can be limited with ultrasound [26,27,28].

Despite the benefits of MRI, there are discussions about its need in diagnosing PAS. MRI is less accessible than ultrasound, as it is available in limited centers, its cost is higher, and it requires longer examination time and specialized expertise for accurate interpretation of the results [29,30]. Furthermore, its necessity is not clear, compared to ultrasound alone, and there are mixed results from recent studies. Dwyer et al. [29] and Elhawary et al. [26] reported no significant difference in sensitivity of diagnosing PAS between MRI and ultrasound, which means that MRI should be used primarily for cases where ultrasound findings are ambiguous, or when detailed mapping of placental invasion is necessary for surgical planning and management [31].

In addition to that, the latest study by Jauniaux et al. [7] advises caution about routine MRI use, emphasizing that while MRI and ultrasound demonstrate similar diagnostic performance when performed by experienced operators, MRI lacks the capability of color Doppler ultrasound to visualize abnormal placental vascular architecture. Moreover, MRI causes a change in diagnosis in more than one-third of cases, but such changes are often incorrect and could potentially lead to inappropriate alterations in clinical management [28,29,32]. Therefore, current international guidelines do not recommend MRI for routine screening of PAS and advise against its use as an adjunct when ultrasound findings already strongly indicate PAS [7].

As a result, MRI should only be used as a complementary tool rather than a routine screening procedure. Its use should be individualized, and it should be performed only in situations where ultrasound findings are inconclusive because of placental location, maternal habitus, the difficulty in determining the extent of invasion into adjacent organs, or when surgical planning requires detailed anatomy. In such circumstances, MRI may provide additional anatomic details useful for surgical planning, although it is not universally required in all cases. Current international guidelines emphasize that management should primarily be guided by high-quality ultrasound and multidisciplinary team planning, with MRI reserved for select situations where further anatomical clarification is expected to influence surgical approach [7,19,25].

## 3. Correlation of Serial Ultrasound Findings with Clinical Outcomes

Apart from its diagnostic use, ultrasonography has become increasingly recognized as a tool for anticipating maternal and neonatal outcomes in pregnancies complicated by PAS. While the diagnostic features and evolving appearance of PAS have been discussed in this review, an important point is how specific ultrasound findings relate to surgical morbidity, intraoperative blood loss, and neonatal complications, especially when assessed serially across gestation [33,34,35,36].

Many studies have shown that certain sonographic features are associated with increased surgical risk. Bhide et al. [37] emphasized that the presence of multiple, irregular placental lacunae, particularly when accompanied by turbulent Doppler flow, correlates strongly with significant operative morbidity. In their study, these findings were connected with increased rates of major hemorrhage, bladder or urological injury, and an increased need for intensive care unit admission. These associations were not limited to deeply invasive PAS but were also noted in cases of accreta with significant vascular remodeling. Thus, this study underscores that the extent of abnormal placental vascularity, rather than the depth of myometrial invasion alone, often affects surgical risk.

Similarly, Tsankova et al. [38] reported that progression of specific ultrasound markers across gestation can escalate surgical complexity. This cohort showed that initially mild features, such as small lacunae or slight myometrial thinning frequently progressed, in the third trimester, into more apparent features of placental invasion. Enlarging lacunae, deepening myometrial defects, and increasing uteroplacental hypervascularity were all observed in serial scans, and these changes often coincided with greater intraoperative blood loss and the need for extended surgical procedures. As a result, these observations highlight the importance and necessity of serial ultrasound assessment during pregnancy, not only for diagnosis, but for ongoing risk assessment and planning.

However, the relationship between ultrasound findings and clinical outcomes is not always straightforward. Jauniaux et al. [7] highlighted the distinction between villous tissue invasion and the broader pattern of vascular remodeling within the uterus and surrounding tissues. Even in cases without full-thickness penetration of the myometrium, extensive vascular channels may develop, and this creates substantial risks during surgical dissection. Ultrasound can provide important information, but it does not predict surgical difficulty with complete accuracy. Therefore, it is very important to integrate ultrasound data with clinical judgment and multidisciplinary expertise.

Beyond maternal morbidity, serial ultrasound findings have implications for neonatal outcomes. Pregnancies with severe ultrasound markers are most commonly planned to deliver early, usually with an elective cesarean delivery at 34–35 + 6 weeks [8], in order to reduce maternal hemorrhagic risk, and, consequently, maternal morbidity. Planned delivery at 36–38 weeks, when no acute complications are present, optimizes neonatal outcomes without increasing maternal morbidity, particularly in centers with multidisciplinary expertise [39,40]. In contrast, emergent deliveries precipitated by bleeding or labor occur at earlier gestations and are associated with greater transfusion needs and worse neonatal outcomes [39]. In addition, the benefit of elective cesarean delivery earlier lies in its capacity to optimize surgical conditions by enabling controlled uterine entry, minimizing placental disruption, and facilitating advanced perioperative measures such as cell salvage, targeted hysterotomy, and coordinated blood bank support, all of which reduce blood loss and maternal morbidity [41]. When delivery is planned before 37 weeks, this approach also permits timely administration of antenatal corticosteroids to decrease neonatal respiratory complications [1]. Compared with expectant management or attempted vaginal delivery, pre-labor cesarean performed in a specialized center is associated with superior maternal and neonatal outcomes, particularly in pregnancies with high-risk ultrasound features [8,41]. Del Negro et al. [42] showed that women with accumulation of severe ultrasound findings delivered earlier, leading to higher rates of neonatal intensive care unit admission and longer hospital stays for newborns. These findings underscore the challenge that clinicians face in optimizing perinatal outcomes for both mother and infant. Thus, the care team should always balance maternal safety against potential neonatal complications associated with prematurity.

In practical clinical management, serial ultrasound findings frequently shape decisions about the timing and setting of delivery. Selby Chacko et al. [43] showed that progressing features in the ultrasound directly influenced perioperative planning. Pregnancies that had progressive changes, such as newly developing bridging vessels, further thinning of the myometrium, or worsening lacunae, were more likely to be referred to tertiary centers, equipped with trained surgical teams, for the delivery. These findings show that monitoring with serial ultrasonography provides the opportunity of taking proactive measures, and it facilitates better preparedness for potentially complex surgeries, including preoperative mobilization of blood products and involvement of urological or vascular surgical specialists when needed. As a result, serial ultrasound surveillance contributes to reducing unexpected surgical emergencies and improving maternal outcomes [44].

In addition, there is consensus that serial ultrasound remains invaluable for guiding management in PAS. Tsankova et al. [38] emphasized that serial assessment, even in cases initially identified as low risk, can find patients who develop more pronounced findings later in pregnancy. Such surveillance enables early referral to specialized centers, surgical planning, and appropriate allocation of resources, with the ultimate goal of reducing maternal and neonatal morbidity.

Nevertheless, challenges remain in the ultrasound assessment of PAS, and one of the most significant is interobserver variability. Bhide et al. [37] demonstrated that specific features, such as prominent lacunae, tend to be more reproducible across observers, but other signs, like subtle myometrial thinning or bladder wall disruption, are more subjective, which causes divergences in interpretation. This variability may affect the diagnostic accuracy, as well as the prognostic value of ultrasound findings. The establishment and implementation of standardized imaging protocols, as well as operator training, are essential in order to enhance consistency and reliability in interpreting these ultrasonographic findings.

Moreover, the correlation between ultrasound findings and clinical outcomes is influenced by additional factors, including the location of the placenta, prior surgical history, and individual anatomical variations. For instance, posterior PAS remains more difficult to evaluate sonographically due to limited acoustic windows, and this potentially reduces the accuracy of ultrasound in predicting surgical risks in these cases [7]. This limitation suggests that even when serial ultrasound is diligently performed, there will remain a small number of patients for whom surgical findings might differ from imaging predictions. Prior surgical history that may affect the outcome includes prior cesarean delivery, prior myomectomy, uterine artery embolization, dilation and curettage, hysteroscopic adhesiolysis, endometrial ablation, operative hysteroscopy, and prior surgical abortion [45]. To this point, there is not enough evidence that serial ultrasound for PAS improves maternal or neonatal outcomes compared with single or limited assessments. Their main benefit is that they offer greater diagnostic certainty, evaluation of risk, and multidisciplinary planning. Although many clinicians perform monthly scans in pregnancies at risk, this is based on expert consensus rather than outcomes from large cohorts [8]. Ultrasound still remains highly accurate for diagnosis, but increased scan frequency beyond what is necessary for diagnosis and surgical planning has not been shown to reduce hemorrhage, hysterectomy, or neonatal morbidity. The primary value of serial imaging is in monitoring placental invasion and supporting delivery planning at experienced centers.

An additional consideration is the psychological impact of serial ultrasound monitoring on patients. Despite repeated imaging offers significant clinical benefits, it may increase maternal anxiety, particularly when new findings emerge suggesting a more severe form of PAS. There is a need for clear communication with patients about the purpose of serial assessments and the implications of changing ultrasound findings, in order to avoid stress and achieve informed shared decision-making.

To summarize, current evidence clearly supports the clinical utility of tracking sonographic changes over time, showing that the integration of serial ultrasound findings into the assessment for PAS might expand further. Such practice enables personalized care pathways, aligning surgical resources with anticipated complexity and improving outcomes for both mother and child. In conclusion, serial ultrasonography is very important to the obstetricians dealing with PAS, as it offers critical insight into the evolving signs. By correlating specific sonographic features with clinical outcomes, serial imaging helps in the prediction of operative challenges, guides the timing and location of delivery, and informs about maternal and neonatal risks. Continued refinement of imaging techniques, standardization of assessments, and integration into comprehensive care models will be essential to fully leverage the prognostic potential of serial ultrasound in managing this complex obstetric condition.

## 4. Scoring Systems and Risk Stratification

Risk stratification in PAS has advanced significantly over recent years, with multiple scoring systems proposed to enhance diagnostic accuracy and inform clinical management, as reliance on a single sonographic marker is often insufficient given their variable sensitivity and specificity. Scoring systems that combine multiple ultrasound markers, and sometimes clinical risk factors, have been developed to overcome these limitations and better predict the severity of placental invasion and potential surgical complexity.

One suggested approach has been to integrate both maternal history and ultrasonographic findings into composite risk models. Gao et al. [46] developed a comprehensive scoring system that combined maternal characteristics, such as parity, prior cesarean sections, and previous uterine curettage, with specific ultrasound markers including placental lacunae, subplacental hypervascularity, placental bulge, and bladder wall interruption. This combined score achieved high diagnostic performance, with an area under the ROC curve of 0.925. Features like a placental bulge and bladder wall interruption had the highest individual point values in Gao’s system, accentuating their strong association with deeply invasive disease.

Mahalingam et al. [47] proposed the Placenta Accreta Scoring System (PASS), which integrates clinical history and MRI findings rather than using ultrasound alone. Their model allocated points for factors such as prior cesarean delivery, placenta previa, loss of the uterine–placental interface, myometrial thinning, intraplacental vascular channels, and focal uterine bulging. Patients were divided into three groups (low, moderate, and high-risk), with the high-risk group having a 90%prevalence of confirmed PAS. This underscores the value of multimodal imaging, especially MRI, when ultrasound results are equivocal or in cases of posterior placenta location.

An important recent contribution to risk stratification comes from Sargent et al. [48], who developed a predictive model using standardized ultrasound descriptors codified by the European Working Group for Abnormally Invasive Placenta. In their prospective cohort of women with anterior low-lying placenta or placenta previa and prior cesarean delivery, they demonstrated that just four ultrasound features, which included the loss of the clear zone, abnormal placental lacunae, placental bulge, and bladder wall interruption, could reliably differentiate between normal placentation, abnormally adherent placenta, and abnormally invasive placenta. Their model achieved a high predictive accuracy with a C-index of 0.901, with strong potential for clinical application. This work is notable for being among the first to link standardized sonographic markers directly to PAS severity as defined by the FIGO classification, offering a practical tool that could be adapted into digital formats for clinical use.

Several ultrasound-only scoring systems have also shown promising diagnostic utility, as the one described by Zhang et al. [49]. They established a scoring system that quantified seven ultrasound features, including placental location, placental thickness, presence or absence of retroplacental space, myometrial thickness, placental lacunae, retroplacental blood flow, and history of cesarean section. Higher total scores correlated strongly with deeper grades of placental invasion, and as a result, this enabled the discrimination between accreta, increta, and percreta cases. Their model provides a pragmatic approach that could be readily implemented in clinical practice, particularly in settings where MRI may not be routinely available. The different scoring systems are presented in Table 2.

Pekar Zlotin et al. [53] conducted a systematic review and meta-analysis evaluating several clinical–sonographic scoring systems. Their analysis confirmed that specific sonographic markers, especially loss of the clear zone, placentation in the lower uterine segment, and presence of placental lacunae, had the highest predictive value for PAS. However, they noted heterogeneity in the thresholds used across studies. They reported pooled sensitivities and specificities above 80% for several integrated sonographic scoring models, supporting their potential use in routine prenatal screening.

However, Jauniaux et al. [7] emphasized that while scoring systems improve risk stratification, they cannot fully predict the complex surgical risks associated with abnormal placental vasculature and pelvic adhesions. They highlighted the importance of interpretation of imaging-based scores alongside clinical factors and that they should be used as part of a multidisciplinary planning process, rather than serving as standalone decision tools. This approach remains essential, particularly in light of the high maternal morbidity associated with unexpected PAS diagnoses at delivery.

Additionally, serial prenatal ultrasound across trimesters enhances prediction of surgical complexity in PAS, though its value depends on timing. First-trimester scans can identify high-risk implantation patterns, but their specificity for severity is limited unless combined with later evaluations. Second- and third-trimester ultrasounds provide greater diagnostic accuracy, as features such as placental lacunae, myometrial thinning, loss of the clear zone, and bladder interface irregularity become clearer and correlate with the need for extensive surgery, including hysterectomy. Transvaginal ultrasound in the third trimester further refines prediction by evaluating lower uterine segment thickness, cervical changes, and vascularity. ACOG, SMFM, and SGO recommend serial imaging at 18–20, 28–30, and 32–34 weeks to support surgical planning and multidisciplinary preparedness, with the greatest predictive value achieved by third-trimester assessments close to delivery [9].

It is clear that certain ultrasound findings consistently emerge as the most predictive markers. These signs, when systematically assessed and combined into scoring algorithms, significantly enhance diagnostic accuracy and enable more precise surgical preparation. Scoring systems and risk stratification models represent an important advancement in the management of PAS, and they offer a structured method for integrating imaging findings and patient history into clinical decision-making. As new studies continue to refine these tools, their widespread adoption, combined with rigorous operator training, minimization of interobserver variability in interpreting ultrasound signs, universal standardization of scoring thresholds and standardized protocols and multidisciplinary care will be essential to improve maternal and neonatal outcomes in this challenging obstetric condition.

## 5. Future Directions

Although significant advances have been made in the diagnosis and management of PAS, important gaps remain that require further research and innovation. A major priority is the standardization of ultrasonographic protocols, not only in definitions of key markers such as placental lacunae, myometrial thinning, and bladder wall disruption, but also in establishing evidence-based guidelines for the optimal timing and frequency of serial scans. Despite consensus efforts, substantial variability persists in how ultrasound features such as placental lacunae, myometrial thinning, and bladder wall disruption are defined and reported [7,37]. Achieving greater uniformity in definitions and imaging techniques will be necessary to improve diagnostic reliability.

Another critical direction is improving interobserver reproducibility. Despite consensus efforts, the interpretation of ultrasonographic features continues to vary substantially between operators. Strategies such as structured reporting, training modules, and consensus-based scoring systems should be systematically evaluated for their ability to improve consistency.

The validation of scoring systems represents another critical future direction. Although several ultrasound-based models have demonstrated promising diagnostic performance, they have been evaluated in single-center cohorts. So, the organization of prospective, multicenter studies are needed to confirm their utility and to define standardized thresholds that can be used globally and help in management decisions across diverse clinical settings.

Further research is needed in order to evaluate the integration of supplemental methods for assessing PAS. MRI remains valuable in selected cases, as mentioned above, but its precise role alongside serial ultrasonography requires clarification. Similarly, biomarkers of abnormal placentation could complement imaging by providing objective measures of disease severity. New technologies such as artificial intelligence (AI) hold significant potential for PAS diagnosis and risk prediction. AI algorithms could support automated detection and quantification of ultrasonographic features, in order to reduce interobserver variability and enhance the consistency of risk assessments. However, development and clinical validation of such tools require very large datasets with high-quality imaging and collaboration across research centers. These innovations, however, require large, high-quality cohorts and rigorous clinical validation before implementation.

The integration of predictive models into digital platforms or mobile applications is another promising direction. These tools could enable real-time risk assessment, even in lower-resource settings, and help in guiding referrals and surgical planning [48]. Ensuring these technologies are evidence-based, user-friendly, and accessible will be critical for safe clinical implementation.

Very important is the refinement of the optimal timing of delivery in PAS. While current recommendations suggest planned delivery between 34 and 36 weeks to mitigate maternal risk, balancing this with the goal of minimizing neonatal morbidity from prematurity, further research is needed to individualize delivery timing based on dynamic risk prediction, potentially by serial ultrasound findings. Serial ultrasound assessments may play an increasingly important role in individualizing delivery timing in the future. Collectively, these areas of research hold significant promise for improving diagnostic precision, surgical planning, and outcomes in pregnancies complicated by PAS.

## 6. Conclusions

Placenta accreta spectrum is a growing obstetric challenge due to increasing cesarean delivery rates and uterine surgery worldwide. Early and accurate prenatal diagnosis is essential for decreasing the substantial maternal and neonatal risks associated with this condition. Serial ultrasonographic evaluation has emerged as an important tool, not only for diagnosing PAS, but also for monitoring its progression, and giving the opportunity of dynamic risk stratification and informed decisions regarding the timing and mode of delivery.

Our review highlights that specific ultrasound markers, such as extensive placental lacunae, myometrial thinning, and loss of the clear zone, can progress over gestation, reflecting disease severity and correlating with clinical outcomes, including operative complexity and perinatal morbidity. While single ultrasound assessments are valuable, serial examinations provide more information about the progression of abnormal placentation. However, variability in the interpretation of sonographic features and the lack of universally accepted scoring systems continue to create challenges to consistent diagnosis and management across diverse clinical settings.

Although MRI remains a useful adjunct in selected cases, especially for posterior placentation or inconclusive ultrasound findings, it is not routinely necessary in all patients suspected of having PAS. The primary reliance remains on ultrasound, which, when performed by skilled operators, offers high sensitivity and specificity for diagnosing PAS.

The integration of scoring systems, although promising, requires further validation through large studies to ensure their reliability and applicability in everyday clinical practice. Emerging technologies, including artificial intelligence and digital risk calculators, offer exciting possibilities for improving diagnostic accuracy and accessibility, particularly in resource-limited environments.

Best practice in the management of PAS relies on high-quality ultrasound as the first-line diagnostic tool, with MRI reserved for equivocal or posterior cases. Serial ultrasonographic assessments may be useful in at-risk pregnancies to monitor progression and risk prediction. Delivery should be planned in specialized centers with established multidisciplinary teams, including obstetric, anesthetic, urologic, and transfusion support, to ensure optimal preparedness. In the absence of acute complications, cesarean delivery, often combined with hysterectomy, should be scheduled at 36 weeks, after the administration of antenatal corticosteroids. Ultimately, care depends on early recognition, individualized management, and comprehensive preoperative planning to minimize maternal and neonatal morbidity. Large prospective cohorts are needed to determine whether serial ultrasonographic assessment improves perinatal outcomes. Such studies are also essential for validating standardized diagnostic criteria and risk prediction models and integrating patient-centered outcomes to optimize care for women affected by PAS.

## Figures and Tables

**Table 1:** Ultrasonographic features of PAS.

Ultrasound Feature	Definition/Sonographic Appearance	Prognostic or Surgical Relevance	Enhanced by Doppler?
Loss of retroplacental clear zone	Disappearance or irregularity of hypoechoic area between placenta and myometrium	Suggests abnormal adherence; early sign of PAS	No
Placental lacunae	Numerous irregular hypoechoic vascular spaces with turbulent flow	Number and size correlate with depth of invasion and surgical complexity	Yes
Myometrial thinning or interruption	Focal or diffuse thinning beneath placenta, <1 mm or complete absence	Predicts increased risk of hemorrhage and uterine wall penetration	No
Placental bulge	Deviation of uterine contour with outward bulging of placental tissue	Suggestive of increta or percreta; may require surgical readiness	No
Bladder wall interruption	Loss of hyperechoic bladder-serosa interface beneath placental bed	Indicates likely bladder invasion (percreta); mandates multidisciplinary care	No
Bridging vessels	Vessels traversing uterine serosa to adjacent structures	Sign of advanced invasion (percreta); surgical complexity likely	Yes
Chaotic subplacental vascularity	Turbulent and disorganized flow at the placenta–myometrium interface	Correlates with deep invasion and risk of massive hemorrhage	Yes
3D Power Doppler hypervascularity	Dense, aberrant vascular networks, especially beyond serosa	Most useful in posterior PAS or suspected bladder invasion	Yes

**Table 2:** Ultrasound-based scoring systems for PAS.

Scoring System/Tool	Key Ultrasound Markers/Features	Clinical Risk Factors Included	Single vs. Serial Assessment Performance	Clinical Focus/Utility
Zhang et al. Scoring System [49]	Placental location, thickness, retroplacental space, myometrial thickness, lacunae, retroplacental blood flow	History of cesarean section	Good diagnostic efficacy; serial improves prediction	Diagnosis, severity, surgical planning
Yang et al. Scoring System [50]	Placental lacunae, loss of clear zone, bladder wall interruption, placental bulge, bridging vessels, myometrial thinning	None	AUC 0.87 for percreta; serial increases predictive value	Staging, prediction of hemorrhage/hysterectomy
Delphi Consensus (ISUOG/SMFM) [51]	Loss of clear zone, myometrial thinning, bladder wall interruption, placental bulge, uterovesical hypervascularity, lacunae, bridging vessels	Prior cesarean/myomectomy	Standardized reporting; serial recommended for high-risk	Consensus on standardized markers
Zheng et al. Scoring System [52]	Maternal risk factors + ultrasound features (lacunae, clear zone, bladder wall, bulge, bridging vessels, myometrial thinning, hypervascularity)	Maternal risk factors	AUC 0.96 for percreta; validated for severity and outcomes	Predicts hemorrhage, hysterectomy
Chong et al. Scoring System [17]	Placental lacunae, loss of clear space, increased retroplacental vascularity, size/number of lacunae	Prior cesarean	Predicts bleeding and hysterectomy; serial improves accuracy	Prognosis, risk stratification
Pekar-Zlotin et al. Scoring System [53]	Anterior low-lying/placenta previa, absent clear space, increased retroplacental vascularity, size/number of lacunae	Prior cesarean	Score increases with risk factors; serial improves grade prediction	Comparison with clinical grading
Aryananda/Adu-Bredu Model [12]	Intracervical hypervascularity, distorted bladder wall, parametrial hypervascularity, LUS remodeling, serosal hypervascularity, uteroplacental vascular remodeling	None	High accuracy for hysterectomy prediction; serial not specified	Distinguishes PAS from scar dehiscence
Watthanasathitnukun et al. [54]	Focal exophytic mass, placental bulge, placental lacunae feeder vessels (2D signs)	None	AUC 0.80 for massive perioperative blood loss; single scan predictive for PBL >2500 mL; serial not specified	Prediction of massive perioperative blood loss in previa/PAS
Cali et al. [55]	Placental lacunae, loss of clear zone, bladder wall interruption, uterovesical hypervascularity, parametrial vascularity	Placenta previa	Staging correlates with EBL, transfusion, operation time, complications; serial staging increases correlation with surgical outcomes	Staging PAS severity and surgical risk in previa cases
Bartels et al. [9]	Abnormal placental lacunae, myometrial thinning, loss of clear space, bladder wall interruption, placental bulge, hypervascularity, bridging vessels	Prior cesarean	Sensitivity 0.84, specificity 0.73 for PAS in third trimester; checklist increases detection in high-risk; serial not specified	Sonographic checklist for PAS detection in high-risk pregnancies
Khandelwal et al. [9]	Placental lacunae, myometrial thinning, hypoechoic zone/bulge, increased flow on color Doppler	None	Markers used for early and late gestation PAS diagnosis; serial evaluation recommended for evolving findings	Standardized imaging and reporting for PAS diagnosis
Skupski et al. [56]	Classic: lacunae, bladder wall interruption, myometrial thinning, hypervascularity; Novel: small lacunae, irregular PMI, vascular PMI, bulge	None	Combined model AUC 0.88; more signs = higher PAS risk; serial increases detection, especially with novel signs	Combined classic and novel markers for improved PAS detection
Fratelli et al. (ADoPAD) [57]	Hypoechogenic space loss, bladder wall interruption, abnormal placental lacunae	Prior cesarean, anterior placenta	Post-test probability for PAS increases with more markers; high specificity; serial third-trimester scans refine risk, especially in previa	Prospective multicenter validation for PAS in previa

AUC: area under the curve, PAS: placenta accreta spectrum, LUS: lower uterine segment, PBL: perioperative blood loss, PMI: placenta–myometrium interface, EBL: estimated blood loss, SMFM: Society for Maternal–Fetal Medicine, ISUOG: International Society of Ultrasound in Obstetrics and Gynecology, ADoPAD: antenatal diagnosis of placenta accreta disorders.

## References

[1] Silver R.M., Barbour K.D. (2015). Placenta Accreta Spectrum. Obstet. Gynecol. Clin. N. Am..

[2] Jauniaux E., Hussein A.M., Einerson B.D., Silver R.M. (2022). Debunking 20th Century Myths and Legends about the Diagnosis of Placenta Accreta Spectrum. Ultrasound Obs. Amp Gyne..

[3] Mohamed E.A., Ahmed R.A., Metwali N.Y., Timraz J.H., Mohamed A., Mansour H.A. (2025). Accuracy of Ultrasound in Prediction of Abnormal Placental Adherence: A Systematic Review and Meta-Analysis. AJOG Glob. Rep..

[4] Jauniaux E., Ayres-de-Campos D., Langhoff-Roos J., Fox K.A., Collins S. (2019). FIGO Placenta Accreta Diagnosis and Management Expert Consensus Panel FIGO Classification for the Clinical Diagnosis of Placenta Accreta Spectrum Disorders. Int. J. Gynecol. Obs..

[5] Collins S.L., Ashcroft A., Braun T., Calda P., Langhoff-Roos J., Morel O., Stefanovic V., Tutschek B., Chantraine F. (2016). On behalf of the European Working Group on Abnormally Invasive Placenta (EW-AIP) Proposal for Standardized Ultrasound Descriptors of Abnormally Invasive Placenta (AIP). Ultrasound Obs. Gyne..

[6] Maged A.M., El-Mazny A., Kamal N., Mahmoud S.I., Fouad M., El-Nassery N., Kotb A., Ragab W.S., Ogila A.I., Metwally A.A. (2023). Diagnostic Accuracy of Ultrasound in the Diagnosis of Placenta Accreta Spectrum: Systematic Review and Meta-Analysis. BMC Pregnancy Childbirth.

[7] Jauniaux E., Aplin J.D., Fox K.A., Afshar Y., Hussein A.M., Jones C.J.P., Burton G.J. (2025). Placenta Accreta Spectrum. Nat. Rev. Dis. Primers.

[8] Cahill A.G., Beigi R., Heine R.P., Silver R.M., Wax J.R. (2018). Placenta Accreta Spectrum. Am. J. Obstet. Gynecol..

[9] Bonanni G., Lopez-Giron M.C., Allen L., Fox K., Silver R.M., Hobson S.R., Nieto-Calvache A.J., Collins S., Wielgos M., Jauniaux E. (2025). Guidelines on Placenta Accreta Spectrum Disorders: A Systematic Review. JAMA Netw. Open.

[10] Vimercati A., Galante A., Fanelli M., Cirignaco F., Vitagliano A., Nicolì P., Tinelli A., Malvasi A., Dellino M., Damiani G.R. (2024). PAS or Not PAS? The Sonographic Assessment of Placenta Accreta Spectrum Disorders and the Clinical Validation of a New Diagnostic and Prognostic Scoring System. J. Imaging.

[11] Jauniaux E., Ayres-de-Campos D. (2018). For the FIGO Placenta Accreta Diagnosis and Management Expert Consensus Panel FIGO Consensus Guidelines on Placenta Accreta Spectrum Disorders: Introduction. Int. J. Gynecol. Obs..

[12] Adu-Bredu T.K., Rijken M.J., Nieto-Calvache A.J., Stefanovic V., Aryananda R.A., Fox K.A., Collins S.L. (2023). The International Society of Placenta Accreta SPECTRUM (IS-PAS) Low- and Middle-Income Countries Working Group A Simple Guide to Ultrasound Screening for Placenta Accreta Spectrum for Improving Detection and Optimizing Management in Resource Limited Settings. Int. J. Gynecol. Obs..

[13] Self A., Cavallaro A., Collins S.L. (2025). Placenta Accreta Spectrum: Imaging and Diagnosis. Obstet. Gynaecol..

[14] Jauniaux E., Bhide A., Kennedy A., Woodward P., Hubinont C., Collins S. (2018). For the FIGO Placenta Accreta Diagnosis and Management Expert Consensus Panel FIGO Consensus Guidelines on Placenta Accreta Spectrum Disorders: Prenatal Diagnosis and Screening. Int. J. Gynecol. Obs..

[15] Calì G., Giambanco L., Puccio G., Forlani F. (2013). Morbidly Adherent Placenta: Evaluation of Ultrasound Diagnostic Criteria and Differentiation of Placenta Accreta from Percreta. Ultrasound Obs. Gyne..

[16] Shih J.C., Jaraquemada J.M.P., Su Y.N., Shyu M.K., Lin C.H., Lin S.Y., Lee C.N. (2009). Role of Three-dimensional Power Doppler in the Antenatal Diagnosis of Placenta Accreta: Comparison with Gray-scale and Color Doppler Techniques. Ultrasound Obs. Gyne..

[17] Chong Y., Zhang A., Wang Y., Chen Y., Zhao Y. (2018). An Ultrasonic Scoring System to Predict the Prognosis of Placenta Accreta: A Prospective Cohort Study. Medicine.

[18] Lerner J.P., Deane S., Timor-Tritsch I.E. (1995). Characterization of Placenta Accreta Using Transvaginal Sonography and Color Doppler Imaging. Ultrasound Obs. Amp Gyne..

[19] D’Antonio F., Iacovella C., Bhide A. (2013). Prenatal Identification of Invasive Placentation Using Ultrasound: Systematic Review and Meta-Analysis: Prenatal Identification of Invasive Placentation. Ultrasound Obstet. Gynecol..

[20] Jauniaux E., Silver R.M., Matsubara S. (2018). The New World of Placenta Accreta Spectrum Disorders. Int. J. Gynecol. Obs..

[21] Jauniaux E., Bunce C., Grønbeck L., Langhoff-Roos J. (2019). Prevalence and Main Outcomes of Placenta Accreta Spectrum: A Systematic Review and Meta-Analysis. Am. J. Obstet. Gynecol..

[22] Shellhaas C.S., Gilbert S., Landon M.B., Varner M.W., Leveno K.J., Hauth J.C., Spong C.Y., Caritis S.N., Wapner R.J., Sorokin Y. (2009). The Frequency and Complication Rates of Hysterectomy Accompanying Cesarean Delivery. Obstet. Gynecol..

[23] Riteau A.-S., Tassin M., Chambon G., Le Vaillant C., De Laveaucoupet J., Quéré M.-P., Joubert M., Prevot S., Philippe H.-J., Benachi A. (2014). Accuracy of Ultrasonography and Magnetic Resonance Imaging in the Diagnosis of Placenta Accreta. PLoS ONE.

[24] Kumar I., Verma A., Ojha R., Shukla R.C., Jain M., Srivastava A. (2017). Invasive Placental Disorders: A Prospective US and MRI Comparative Analysis. Acta Radiol..

[25] Chen Q., Shen K., Wu Y., Wei J., Huang J., Pei C. (2025). Advances in Prenatal Diagnosis of Placenta Accreta Spectrum. Medicina.

[26] Elhawary T.M., Dabees N.L., Youssef M.A. (2013). Diagnostic Value of Ultrasonography and Magnetic Resonance Imaging in Pregnant Women at Risk for Placenta Accreta. J. Matern.-Fetal Neonatal Med..

[27] Guo P., Wu Y., Yuan X., Wan Z. (2021). Clinical Diagnostic Value and Analysis of MRI Combined with Ultrasound in Prenatal Pernicious Placenta Previa with Placenta Accreta. Ann. Palliat. Med..

[28] Lopes E.S., Feitosa F.E.D.L., Brazil A.V., De Castro J.D.V., Da Costa J.I.F., Araujo Júnior E., Peixoto A.B., Carvalho F.H.C. (2019). Assessment of Sensitivity and Specificity of Ultrasound and Magnetic Resonance Imaging in the Diagnosis of Placenta Accreta. Rev. Bras. Ginecol. Obs..

[29] Dwyer B. (2009). Prenatal Diagnosis of Placenta Accreta: Sonography or Magnetic Resonance Imaging?. Yearb. Obstet. Gynecol. Women’s Health.

[30] Hamisa M., Mashaly E., Fathy S., Tawfeek A. (2015). Role of Doppler US and MRI in Diagnosis of Placenta Accreta. Alex. J. Med..

[31] Patel-Lippmann K.K., Planz V.B., Phillips C.H., Ohlendorf J.M., Zuckerwise L.C., Moshiri M. (2023). Placenta Accreta Spectrum Disorders: Update and Pictorial Review of the SAR-ESUR Joint Consensus Statement for MRI. RadioGraphics.

[32] D’Antonio F., Iacovella C., Palacios-Jaraquemada J., Bruno C.H., Manzoli L., Bhide A. (2014). Prenatal Identification of Invasive Placentation Using Magnetic Resonance Imaging: Systematic Review and Meta-Analysis. Ultrasound Obs. Gynecol..

[33] Bailit J.L., Grobman W.A., Rice M.M., Reddy U.M., Wapner R.J., Varner M.W., Leveno K.J., Iams J.D., Tita A.T.N., Saade G. (2015). Morbidly Adherent Placenta Treatments and Outcomes. Obstet. Gynecol..

[34] Feng X., Mao X., Zhao J. (2024). Clinical Characteristics, Prenatal Diagnosis and Outcomes of Placenta Accreta Spectrum in Different Placental Locations: A Retrospective Cohort Study. Int. J. Women’s Health.

[35] Jauniaux E., Kingdom J.C., Silver R.M. (2021). A Comparison of Recent Guidelines in the Diagnosis and Management of Placenta Accreta Spectrum Disorders. Best Pract. Res. Clin. Obstet. Gynaecol..

[36] Jauniaux E., Hecht J.L., Elbarmelgy R.A., Elbarmelgy R.M., Thabet M.M., Hussein A.M. (2022). Searching for Placenta Percreta: A Prospective Cohort and Systematic Review of Case Reports. Am. J. Obstet. Gynecol..

[37] Bhide A., Hussein A.M., Elbarmelgy R.M., Elbarmelgy R.A., Thabet M.M., Jauniaux E. (2023). Assessment of Ultrasound Features of Placenta Accreta Spectrum in Women at High Risk: Association with Outcome and Interobserver Concordance. Ultrasound Obs. Amp Gyne..

[38] Tsankova M., Kirkova M., Geshev N. (2023). Placenta Accreta Spectrum: Ultrasound Diagnosis and Clinical Correlation. Biotechnol. Biotechnol. Equip..

[39] Thang N.M., Anh N.T.H., Thanh P.H., Linh P.T., Cuong T.D. (2021). Emergent versus Planned Delivery in Patients with Placenta Accreta Spectrum Disorders: A Retrospective Study. Medicine.

[40] Rosenthal E.A., White A., Lafferty A.K., Pruszynski J.E., Spong C.Y., Herrera C.L. (2025). Delivery Timing of Placenta Accreta Spectrum: Later Is Feasible. Am. J. Obs. Gynecol..

[41] Gilner J.B., Deshmukh U. (2025). Evidence-Based Perioperative Management of Placenta Accreta Spectrum Disorder. Obs. Gynecol..

[42] Del Negro V., Aleksa N., Galli C., Ciminello E., Derme M., Vena F., Muzii L., Piccioni M.G. (2020). Ultrasonographic Diagnosis of Placenta Accreta Spectrum (PAS) Disorder: Ideation of an Ultrasonographic Score and Correlation with Surgical and Neonatal Outcomes. Diagnostics.

[43] Selby Chacko K., AlSubeaei R.S., Sunil Nair S., Khalil Kazi N., Jeddy R. (2025). Maternal and Clinical Outcomes of Placenta Accreta Spectrum: Insights from a Retrospective Study in Bahrain. Life.

[44] Jauniaux E., Collins S.L., Jurkovic D., Burton G.J. (2016). Accreta Placentation: A Systematic Review of Prenatal Ultrasound Imaging and Grading of Villous Invasiveness. Am. J. Obstet. Gynecol..

[45] American College of Obstetricians and Gynecologists, Society for Maternal-Fetal Medicine (2018). Obstetric Care Consensus No. 7: Placenta Accreta Spectrum. Obstet Gynecol..

[46] Gao Y., Gao X., Cai J., Han F., Xu G., Zhang X., Zhang T., Yu L. (2021). Prediction of Placenta Accreta Spectrum by a Scoring System Based on Maternal Characteristics Combined with Ultrasonographic Features. Taiwan. J. Obstet. Gynecol..

[47] Mahalingam H.V., Rangasami R., Premkumar J., Chandrasekar A. (2021). Placenta Accreta Scoring System (PASS)—Assessment of a Simplified Clinico-Radiological Scoring System for Antenatal Diagnosis of Placenta Accreta. Egypt. J. Radiol. Nucl. Med..

[48] Sargent W., Gerry S., Collins S.L. (2023). A Risk-Prediction Model for Placenta Accreta Spectrum Severity From Standardized Ultrasound Markers. Ultrasound Med. Biol..

[49] Zhang J., Li H., Feng D., Wu J., Wang Z., Feng F. (2023). Ultrasound Scoring System for Prenatal Diagnosis of Placenta Accreta Spectrum. BMC Pregnancy Childbirth.

[50] Yang X., Zheng W., Yan J., Yang H. (2022). Comparison between Placenta Accreta Scoring System, Ultrasound Staging, and Clinical Classification. Medicine.

[51] Jauniaux E., D’Antonio F., Bhide A., Prefumo F., Silver R.M., Hussein A.M., Shainker S.A., Chantraine F., Alfirevic Z. (2023). Delphi consensus expert panel Modified Delphi Study of Ultrasound Signs Associated with Placenta Accreta Spectrum. Ultrasound Obs. Gynecol..

[52] Zheng W., Zhang H., Ma J., Dou R., Zhao X., Yan J., Yang H. (2022). Validation of a Scoring System for Prediction of Obstetric Complications in Placenta Accreta Spectrum Disorders. J. Matern.-Fetal Neonatal Med..

[53] Pekar Zlotin M., Sharabi-Nov A., Meiri H., Revivo P.E., Melcer Y., Maymon R., Jauniaux E. (2024). Clinical-Sonographic Scores for the Screening of Placenta Accreta Spectrum: A Systematic Review and Meta-Analysis. Am. J. Obstet. Gynecol. MFM.

[54] Watthanasathitnukun W., Pranpanus S., Petpichetchian C. (2022). Two-Dimensional Ultrasound Signs as Predictive Markers of Massive Peri-Operative Blood Loss in Placenta Previa Suspicious for Placenta Accreta Spectrum (PAS) Disorder. PLoS ONE.

[55] Cali G., Forlani F., Lees C., Timor-Tritsch I., Palacios-Jaraquemada J., Dall’Asta A., Bhide A., Flacco M.E., Manzoli L., Labate F. (2019). Prenatal Ultrasound Staging System for Placenta Accreta Spectrum Disorders. Ultrasound Obs. Gynecol..

[56] Skupski D.W., Duzyj C.M., Scholl J., Perez-Delboy A., Ruhstaller K., Plante L.A., Hart L.A., Palomares K.T.S., Ajemian B., Rosen T. (2022). Evaluation of Classic and Novel Ultrasound Signs of Placenta Accreta Spectrum. Ultrasound Obs. Gynecol..

[57] Fratelli N., Prefumo F., Maggi C., Cavalli C., Sciarrone A., Garofalo A., Viora E., Vergani P., Ornaghi S., Betti M. (2022). Third-Trimester Ultrasound for Antenatal Diagnosis of Placenta Accreta Spectrum in Women with Placenta Previa: Results from the ADoPAD Study. Ultrasound Obs. Gynecol..

